# It’s not about the capture, it’s about what we can learn”: a qualitative study of experts’ opinions and experiences regarding the use of wearable sensors to measure gait and physical activity

**DOI:** 10.1186/s12984-021-00874-8

**Published:** 2021-05-11

**Authors:** Alison Keogh, Kristin Taraldsen, Brian Caulfield, Beatrix Vereijken

**Affiliations:** 1 UCD School of Public Health, Physiotherapy and Sports Science, UCD, Dublin, Ireland; 2 Insight Centre for Data Analytics, UCD, 3rd Floor, O’Brien Science Centre East, Belfield, Dublin, Ireland; 3 Department of Neuromedicine and Movement Science, NTNU, Trondheim, Norway

**Keywords:** Wearable devices, Acceptability, Remote monitoring, Qualitative, Accelerometry

## Abstract

**Background:**

The use of wearable sensor technology to collect patient health data, such as gait and physical activity, offers the potential to transform healthcare research. To maximise the use of wearable devices in practice, it is important that they are usable by, and offer value to, all stakeholders. Although previous research has explored participants’ opinions of devices, to date, limited studies have explored the experiences and opinions of the researchers who use and implement them. Researchers offer a unique insight into wearable devices as they may have access to multiple devices and cohorts, and thus gain a thorough understanding as to how and where this area needs to progress. Therefore, the aim of this study was to explore the experiences and opinions of researchers from academic, industry and clinical contexts, in the use of wearable devices to measure gait and physical activity.

**Methods:**

Twenty professionals with experience using wearable devices in research were recruited from academic, industry and clinical backgrounds. Independent, semi-structured interviews were conducted, audio-recorded and transcribed. Transcribed texts were analysed using inductive thematic analysis.

**Results:**

Five themes were identified: (1) The positives and negatives of using wearable devices in research, (2) The routine implementation of wearable devices into research and clinical practice, (3) The importance of compromise in protocols, (4) Securing good quality data, and (5) A paradigm shift. Researchers overwhelmingly supported the use of wearable sensor technology due to the insights that they may provide. Though barriers remain, researchers were pragmatic towards these, believing that there is a paradigm shift happening in this area of research that ultimately requires mistakes and significant volumes of further research to allow it to progress.

**Conclusions:**

Multiple barriers to the use of wearable devices in research and clinical practice remain, including data management and clear clinical utility. However, researchers strongly believe that the potential benefit of these devices to support and create new clinical insights for patient care, is greater than any current barrier. Multi-disciplinary research integrating the expertise of both academia, industry and clinicians is a fundamental necessity to further develop wearable devices and protocols that match the varied needs of all stakeholders.

**Supplementary Information:**

The online version contains supplementary material available at 10.1186/s12984-021-00874-8.

## Background

The use of wearable sensor technology to track health behaviours and outcomes of care, using measures of physical activity and gait, has exponentially grown in recent years. Technological advancements have led to the development of smaller, less obtrusive wearable devices that can be used for both recreational and medical purposes, where benefits include the ability to remotely and objectively monitor health behaviours in the context of people’s own lives [1, 2]. In response, the World Health Organisation (WHO) recently created a Digital Health Strategy which recognises the opportunities that exist for digital health, including the ubiquitous presence of wearable devices, to enable improved health coverage and well-being globally [3]. For this to happen, digital health must be accessible, reliable, safe and sustainable [3].

The promise of wearable sensor technology is that it will help transform healthcare by providing access to real-world, objective data [4–6], yet to do this, wearable devices need to meet the needs of all stakeholders who will interact with them. Critical to this is that wearable devices are usable, not only for the participants who will wear them, but also for the researchers and clinicians who implement them, and who ultimately will drive their continued use in various contexts. The International Organization for Standardization (ISO) defines usability as “the effectiveness, efficiency, and satisfaction with which specified users achieve specified goals in particular environments” [7]. It has been suggested that for wearable devices to be accepted, they must be easy to wear, easy to use, affordable, contain relevant functionality and be aesthetically pleasing [8–10]. However, although a number of studies have evaluated participants’ perceptions of various wearable devices for gait or physical activity [11–16], to date, the experiences of researchers who use the data provided by them to address research questions, or assist in clinical decision making, is rarely assessed. Usability should not only be focused on those who wear them, but also those who interact with them to collect and analyse data.

A small number of studies have investigated the use of wearable devices by clinicians [1, 17–19], where barriers such as increased workloads, difficulty handling large volumes of data, and an inability to integrate data into health records have been highlighted. However, typically, these studies have focused on either a single clinical cohort or a single device. Furthermore, to date, no study has evaluated wearable sensor technology from the perspective of researchers who use them. Therefore, there is a wealth of potential learnings and experiences that remains untapped. Specifically, it is important to understand how researchers view the use of wearable devices in healthcare, their perceived barriers and facilitators, the learnings they have gained from participants, and the usability factors that researchers perceive to be the most important or influential when selecting a wearable device for a study. Given the rapid evolution of wearable sensor technology, it is necessary to understand the views of all stakeholders, so that clinically relevant, useful, validated and usable wearable devices can be developed for everyone who needs to interact with them. Therefore, the aim of this study was to explore the experiences and opinions of researchers from academic, industry and clinical contexts, in the use of wearable devices that measure gait and physical activity, across a range of clinical cohorts and ages.

## Methods

### Study design

Ethical approval for this cross-sectional, qualitative study was granted by the Human Ethics Board of the local research institution, University College Dublin (LS-E-19-122-Keogh-Caulfield).

### Participants

Twenty researchers were recruited using purposive, convenience sampling from the Mobilise-D consortium [20]. Mobilise-D is a five-year EU-IMI project that aims to produce validated and accepted digital mobility outcomes to monitor daily life gait of people with different mobility problems [20]. The consortium consists of over 150 professionals from universities, hospitals and industry, with various levels of technical and clinical experience. Members of the consortium were contacted through email inviting them to participate in this study. To participate, members needed to be able to speak English, have personal experience of using wearable devices to collect patient data, and consent to have their interview recorded. All interested participants were provided with information regarding the study procedures and were invited to take part in a single semi-structured interview at a suitable time.

Participants came from a variety of backgrounds including clinical, engineering, sports science and computer science (Table 1). Their experience with wearable devices ranged from one year to 20. Researchers had experience with a range of wearables from one to up to 11 devices.

**Table 1:** Participant details

Variable	
Gender	
Male (n = ; %)	7 (35.0%)
Female (n = ; %)	13 (65.0%)
Professional role	
*Academic*	
Clinical academic	2 (10.0%)
Post-doctoral researcher	3 (15.0%)
Professor	7 (35.0%)
Researcher^a^	5 (25.0%)
*Clinician (full-time)*	1 (5.0%)
*Industry based*	2 (10.0%)
Country of work	
Belgium	1 (5.0%)
Germany	2 (10.0%)
Ireland	2 (10.0%)
Israel	1 (5.0%)
Italy	1 (5.0%)
Norway	3 (15.0%)
Spain	2 (10.0%)
Switzerland	1 (5.0%)
United Kingdom	7 (35.0%)
Years in research (median, IQR; min–max)	11.5 (10.5; 4–31)
Years experience with wearable devices (median, IQR; min–max)	9.0 (7.0; 1–20)
Background area of study (prior to current role)	
Biomedical science	2 (10.0%)
Computer science	1 (5.0%)
Doctor	4 (20.0%)
Engineering	3 (15.0%)
Information technology	1 (5.0%)
Physiology and/or sport and movement science	4 (20.0%)
Physiotherapy	5 (25.0%)
A list of devices/manufacturers that researchers had experience with^b^	
Actibelt; Activpal; Actigraph; Axivity; Biovotion; GaitUp; Hexoskin; Fitbit; Mc10; McRoberts; Movisense; Noraxon; Philips; SenseEye; Sensewear; Shimmer; Strive; Spire, Withings	

^a^Researcher denotes senior researchers, project co-ordinators, and/or research associates; ^b^list is not exhaustive as not all researchers could remember all devices they had used, therefore devices are listed by name only and not how many researchers mentioned them, so as to avoid any suggestion of device popularity

### Protocol

The interview topic guide was developed using the Technology Acceptance Model (TAM) as a theoretical guide [21]. This model posits that technology is adopted when it is easy to use and is perceived to be useful to participants. Interviews were semi-structured in nature. Three main questions were included which related to participants’ experiences of wearables, including the barriers and facilitators of their use (Additional file 1). If required, a number of potential prompts were included in the topic guide, however the interviewer was instructed to be led by the experiences of each participant independently. Interviews were conducted in English and took place using online video calling software between September and November 2019. Interviews were conducted by AK, a female post-doctoral researcher with a background in physiotherapy and previous publications in qualitative research methods. Interviews lasted between 26 and 64 min, with a median time of 38 min. Recruitment ceased once data saturation was reached. This was determined by AK, based on whether new information was gained from further interviews. All interviews were audio-recorded and transcribed verbatim.

### Analysis

Interviews were coded using an inductive thematic analysis from a realist perspective, whereby it was assumed that the interviews reported the experiences, meanings and reality of the interviewees [22]. No specific theoretical constructs were used to analyse the transcribed texts [22]. However, codes were developed early in the analysis, using the extensive experience of the coders in this area. Thus, a ‘codebook’ method of analysis was used whereby a rich thematic description was sought so that predominant and important themes throughout the full dataset were identified through merging and interpreting the relationship between codes [23].

**Fig. 1:**
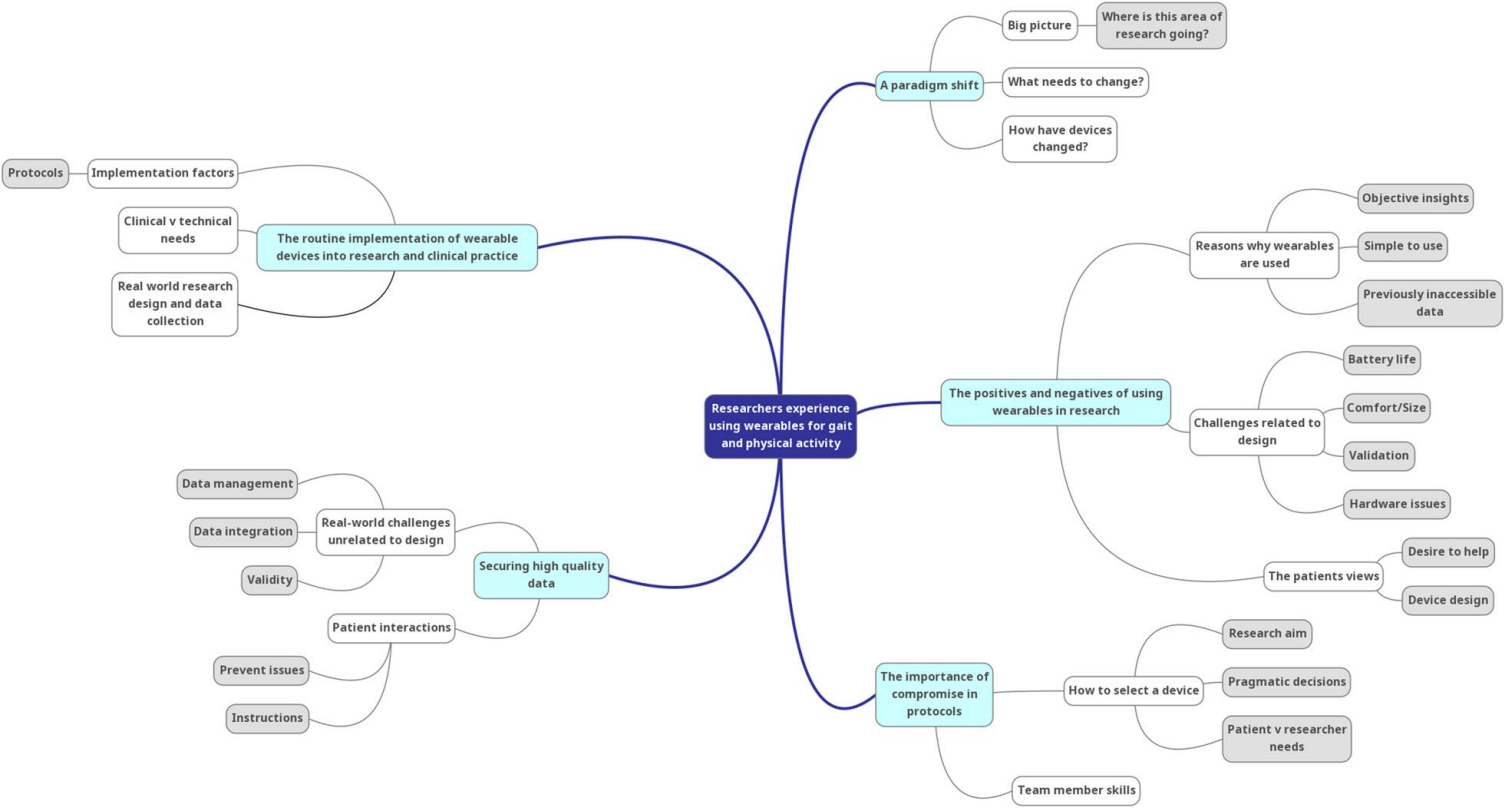
Representative mind map

Interviews were coded by three researchers, AK, BV and KT, following the suggested protocol of Braun and Clarke [22]. BV is a female Professor of Human Movement Science. KT is a female physiotherapist with a PhD in Clinical Medicine. All three coders are members of the Mobilise-D consortium. Coding was completed manually. The interviewer (AK) familiarised herself with the full data set by reading all transcripts twice. An initial coding book was developed and sent to BV and KT for review until agreement was reached (Table 2). Using this developed coding book (Additional file 2) each text was coded by AK (n = 20), while BV and KT coded five texts each, thus 50% (n = 10) of texts were double rated (Table 2). Inter-rater reliability was established using percentage agreement. Agreement was above 80%, indicating excellent reliability of coding. Codes were then collated into potential themes and subthemes. These themes were subsequently refined using mind maps and discussion between all three raters (Fig. 1). Compelling extracts from the interviews were identified to ensure that the themes represented the data set before a final set of themes was agreed upon.

**Table 2:** Coding process

Step 1	Rater 1 read all de-identified texts twice (AK)
Familiarisation	Notes taken to manually develop an initial coding book
	Draft 1 developed by Rater 1 (AK)
Step 2 Coding book development	Draft 1 of coding book, plus a single, de-identified text, sent to Rater 2 (KT)
	Coding book refined by AK and KT
	Draft 2 of coding book sent to Rater 3 (BV)
	Another de-identified text emailed to all coders All raters manually coded this text with Draft 2
	Online meeting held between raters to refine coding book through discussion
	Final version of coding book confirmed
Step 3 Final coding process	Final version of coding book, plus remaining de-identified texts emailed to all raters Final version of coding book used to manually code all texts (Additional file 2)
	Rater 1 (AK) coded all texts (n = 20)
	Raters 2 and 3 (KT and BV) coded four additional texts each (n = 8)

## Results

Five themes were identified in the analysis: (1) The positives and negatives of using wearable devices in research, (2) The routine implementation of wearable devices into research and clinical practice, (3) The importance of compromise in protocols, (4) Securing good quality data, and (5) A paradigm shift. Supporting quotations are provided in Tables 3, 4, 5, 6, 7.

**Table 3:** Supporting quotations for Theme 1 ‘the positives and negatives of using wearable devices in research’

Theme 1: The positives and negatives of using wearable devices in research
“I’ve seen myself from my own experience in a number of trials is that the more ill the individuals are, or the more let’s say, not more degenerative the disease or the more advanced it is or whatever, they feel like if they thought that something could benefit this, even though we always explain that you are not going to directly benefit from this but this research might benefit people in similar situations, then they do feel, like willing to participate.”—Participant 6, Male, Academic
“So, the positives with wearables in general is that you can use them wherever you want. So if you are not too keen about using magnetometers then what you can do is you can literally use them anywhere and they are so small that you can, that you can, eh put them on shoes and on human bodies without them being invasive or annoying to them, to patients and also to eh to the people that are doing the experiment.”—Participant 1, Male, Academic
“Now I know, okay, another problem is that we, so we do not know a lot about certain diagnosis and how they perform in a way in the home environment. We know as clinicians a lot about how people perform in the hospital and the doctor's practice but we do not know what they do in the home environment. So we are not aware of many symptoms that may exist and do exist but we have never seen them, we have never understood them as medical professionals and the questionnaires, they do not help us with that because the questionnaires have all to be designed by medical doctors and neuropsychologists etc. who have seen the patients in the professional environment. So they cannot ask for these symptoms because only if patients have an understanding of the symptoms by themselves. In Parkinson’s Disease for example, I have the impression that we see entirely new symptoms with this new trend in getting into the home environment which we have never seen in the hospital. This is also something which is a real paradigm shift or paradigm change in the management of diseases for doctors. And you know that it means also for doctors a new understanding of how we manage our patients and we are not yet there with this paradigm shift with regard to how we have to get use of this new technology. It is not only the technology which we need to have a better understanding of something, you need the people, that they accept that there is something new around and that it doesn't solve everything.”—Participant 16, Male, Academic
“But that is for me where it is a game changer, the fact that you can really, it’s a discovery, it is what it allows you to do and you can measure things on 100 s of patients rather than on 10 patients and that is still on the clinical research side. On the real life monitoring as I said for me, is this… I believe in the power of going out of the lab and seeing the other dimension, the one that you don't see in the lab. So what does my patient do when they go home?”—Participant 5, Female, Academic
“And I think one challenge is really combining them all, so if you could help ahead of time to think through, ok how can we combine the different output files so that we can get, maximise the analysis that we can do on these files. I think that would be something that would be really helpful.”—Participant 3, Female, Academic
“People don't realise always that when we say you can take it off after one week that they are done and they throw it away so they don't remember to send it to us or bring it back to us.”—Participant 8, Female, Academic

**Table 4:** Supporting quotations for Theme 2 ‘the routine implementation of wearables into research and clinical practice

Theme 2: the routine implementation of wearables into research and clinical practice
“That is indeed a problem because I am the reviewer of many papers and I see … of fantastic technical backbone but I see that it does not make sense from a clinical point of view, what they want to measure. Or maybe it is not useful but it does not make sense with regard to quality of life or whatever and the other way around. So when clinicians are measuring it is often obvious that the technology background is missing. So at least in my view the most important aspect is that the relevant stakeholders are sitting together, they try to understand each other's language and everyone is bringing every argument why something should work or should not work and why it is important or why it is not important.”—Participant 16, Male, Academic
“An interesting part in this sense is also the clinicians' expectations because there are so many times, I mean of course the clinicians I interact with are involved in research somehow and they are a different species but what happens to me is when I talk to a lot of consultants in the hospital for example they come to me and they are like, fantastic let's go and look at what happens to the patients in the house. And then when you ask them why, so what is the information that you want? They haven’t a clue. So they know that they want something but what will they do with that information? To me it is not that clear”—Participant 5, Female, Academic
“Certainly, for COPD which is my area, home monitoring so far for clinical purposes has been a complete dud. Eh well because the things that you can measure, oxygen saturation, heart rate, spirometry those kind of things are just, do not produce, they don’t influence clinical decision making in a positive way..… it’s just that the current technology and the current framework just isn’t there”—Participant 19, Male, Academic
“I think typically we are less, most of the human factors reasons we end up losing data not on the patient side but on the investigator side where you know….that’s another thing. You could arguably say that that’s one of the potential pitfalls of this, you know, the ubiquity of sensors, and it’s that when you bring a new researcher into a motion capture laboratory and they see the CODA system and they go wow that’s amazing. I’m going to have to get training so I can use that. I’m not going to be able to just come in, turn it on and figure it out. Whereas when they see a sensor they’re often like oh well that’s pretty easy you know, and frequently it’s not. Frequently there are you know again we’ve had so many times where somebody has put Shimmer on upside down, we’ve had so many times where somebody has forgotten to calibrate a sensor or you know things like that, they didn’t remember that the last person that used it before them was doing some jumping studies and the accelerometer range was completely different than they needed.”—Participant 4, Male, Academic
“That I don't know actually because I am not that software engineer so sometimes you upload the data directly to their service and from a clinical point of view you then look into the reports as soon as they are processed. Also because we are not that proficient with the scripting and the algorithms we tried to stay away from that as well, because you don't have enough background, you don't trust yourself fully enough.”—Participant 14, Female, Clinician
“No I’ve got a multi-disciplinary team, I’ve got 20 people in my group, half of which will be engineers of different engineering disciplines as well as computer scientists, mathematicians, clinicians, movement scientists. And I deliberately set up my team because I wanted to work with wearable sensors so that was a very deliberate choice.”—Participant 12, Female, Academic
“And of course from a clinical point of view sometimes you think that is not that difficult, you just turn your signal upside down or make the absolute values and then everything is going but of course from an engineering point of view that is not always the case”—Participant 13, Male, Academic

**Table 5:** Supporting quotations for Theme 3 ‘the importance of compromise in protocols’

Theme 3: the importance of compromise in protocols
“So the arm ones weren’t as accurate as the trunk ones ehm, so it comes back because you know there are things that you can wear on your wrist, eh which is more natural as people are used to wearing watches. So eh, you know in some situations for much more prolonged wearing that might be preferable but you know you’re going to have to be careful because you’re going to be losing some sort of precision particularly around walking”—Participant 19, Male, Academic
“What can we do to minimise the burden to the participant? But it has to be counter balanced with, we want data of this quality so we’re clear of what we need from the device so it’s that balancing act. And in using the devices that we have, we know that they’re not perfect but we have chosen to eh, we can accept the compromise and still get data that. Gives us good information.”—Participant 12, Female, Academic
“Even if it’s some small vendor who is not part of the preferred vendor list of the company and we try to find the best fit. If that’s not possible, too expensive, you know too many GDPR issues, we sort of downgrade and say ok what’s the bare minimum we need and sort of to be, it’s almost a bit cynical but in the end its very often ‘ok, we’ll just stick an Actigraph to the non-dominant wrist’. We find ok at the beginning you said you wanted specific gait parameters, you can’t do that if you stick an Actigraph to the non-dominant wrist, eh so either we do it properly or we don’t do it at all and I think that’s also important that if we deviate too much from what we want we have to say no otherwise we’ll do too much harm to ourselves.”—Participant 17, Male, Industry
“If we are just talking about research and we need to push through a high number of patients while all the patients are in the clinic, then I would go for an easy system that is easy to use for the people that do the data collection so in our case medical students for example and then that would be a different system so I cannot really say which system is best because it really depends on what you want. This is why in XX for example we often end up with multiple systems that we are using for multiple things so if we are going for home assessment we are using a different brand than we are using for an in-clinic assessment.”—Participant 1, Male, Academic

**Table 6:** Supporting quotations for Theme 4 ‘securing good quality data’

Securing good quality data
“I have an information sheet of how to wear and then I go through it with the patient, I go through the importance of it, why we use it, why we need it for seven days, those sorts of things.”—Participant 8, Female, Academic
“I think we have better compliance because we’ve got pretty good protocols and data quality checking methods and very robust processes to follow up trial participants or the assessors that are putting the devices on pretty quickly so that we don’t leave six months of data and then go oh by the way we better have a look at that data and see if it’s ok [laughs]. We’re onto it straight away.”—Participant 12, Female, Academic
“What we usually do in our clinical studies is we make a very easy to use booklet with figures and then also the first time is that we ourselves apply the systems and we tell in this case if it is the therapist or the patient on what they should be aware of. And we also point them in the booklet that they get so the small brochure for instance where they can find this information. And then next we let them apply everything and just see if they do it correctly and if not then we correct them. Because sometimes you don't think to mention something and you see the patient is doing that and you can intervene.”—Participant 13, Male, Academic
“The friendly trials for us are really just a confirmation that what we’re doing is you know, is good, has sound logic behind it and in practice it could work.”—Participant 6, Male, Academic
“The challenge is that there are no guidance on what measures to use. So there is a great variability in reported measures so it is not easy to compare between different studies. And that is a challenge I think.”—Participant 11, Female, Researcher
“There’s no standard”—Participant 4, Male, Academic
“And also if something is going to work it’s probably going to be about integrating different measures. So if you measure how much someone is breathing and how much they’re moving you can pick up different patterns of, you know if someone is keeping still and breathing more that’s bad, if someone is keeping still and not breathing they might have just decided that they’re going to have a Netflix binge. Eh so it’s you know, so far we haven’t got the sort of level of integration, it’s monitoring but not what we need.”—Participant 19, Male, Academic
“Yeah because we did validation study in frail populations, we had discussions also with the company on it so what we decided, when we reported outcome in hip pressure patients, for example we only reported by time and we didn't distinguish between gait and standing because we couldn't detect the gait well enough.”– Participant 9, Female, Academic
“Well yes misclassification of activities. I mean I didn’t do it because I was just designing the protocol, but I am aware from literature that misclassification of data is a big problem.”—Participant 2, Female, Academic
“We get the raw data….but again it’s a black box. Because they are all very sweet and nice guys but all their validation things if you dig a bit deeper, are very obscure. So I’ve cited this one, they’ve cited me and then we’ve cited each other and this is our validation so it’s like ahhhh cool but I don’t really trust you.”—Participant 1, Male, Academic
“I suppose if the patient could see something, if they could see what they are recording or that sort of thing I think patients would be more likely to wear it then, if it had a step counter in the front. Then I think it might encourage them to walk more than they normally do or whatever. But I think that probably would encourage the patients to wear it more.”—Participant 14, Female, Clinician
“Because patients really want to, this is my experience, they want to be able to understand their own conditions, particularly if they’ve got chronic, long term health conditions. They really welcome the feedback, you know the feedback that they get is that they see the benefits of these tools. So, and anything that they think can help the doctor, or the nurse or the physiotherapist have better information, that also helps them and might help with their medication adjustment and all the rest of it. They really are sensible actually and see the benefits.”—Participant 12, Female, Academic
“I think sometimes people can overegg burden a little. You know my experience is that most people who are getting involved in a research study, they’re doing it because they want to help. They’re not doing it out of a sense of obligation or entitlement. You’re generally working with people who are a little bit more giving, that’s the reason they’re there. And, I think people who are involved in studies would expect to have to have some level of active involvement, it’s not all passive.”—Participant 4, Male, Academic
“So I think one of the docmarks that we had a while ago was do not influence the patient or don’t show the number of steps tracked or don’t show anything just stick a wearable to them. Now, that has changed. I think that you need to give feedback to encourage the patient to continue wearing the wearable. It doesn’t mean that we have to give details or statistics on performance, even just wear time for example. So feedback and sort of monitoring of wearing is in my opinion very important. If the patient wants to know and wants to participate you know if you just stick a black box to the patient who doesn’t interact with it then I think that is not a good idea.”—Participant 17, Male, Industry

**Table 7:** Supporting quotations for Theme 5 ‘a paradigm shift’

Theme 5: A paradigm shift
“Eh, from the clinical operations perspective at the moment. So let’s say the aim is to make our trials better in terms of faster, more precise, getting better readouts and cheaper. At the moment the reality is a bit different. At the moment they make it slower and have additional patient and in-site burden and makes them more expensive. So we are still sort of working towards the benefits of it. I don’t know, or I wouldn’t say that its necessarily a downside but if this situation continues too long then patients or maybe stakeholder will sort of lose interest sand it will have a bad effect on the field itself. So I think it needs, sort of from the pharma perspective we do need to show very clear impact either on cost, operations or quality of endpoints.”—Participant 17, Male, Industry
“I think that there has been a massive, I mean for me it has been interesting to observe in the sense that coming from the technical background, seeing the uptake before these products were actually ready to be out there, for me was the most interesting part of the story. And somehow it is something that reminded me of what happened with motion capture back in the days. Everyone started having these labs where they think that it was just something that you started using and you had all the data that you collected.”—Participant 5, Female, Academic
“In my mind the early stages, more of the focus was on the hardware, and then the ehm, after some of the hardware questions kind of started to settle down a bit then it became more about studies that were aimed at validating the capacity of the IMUs or activity sensors to, in particular if we take the IMUs, it’s kind of moved through different ages. It’s gone from that age of ok, can we eh, lets develop on the hardware level first, then let’s see how well we can measure existing gait measures so like spatiotemporal measures of gait etc. in a controlled laboratory environment, to then gradually looking at ok, lets now that we’ve figured out how we can validate it against gold standard for measuring spatiotemporal parameters, now can we see if we can identify any differences associated with this clinical group or that clinical group so we are comparing the groups to the norm, then there’s that evolution into I suppose seeing whether or not we can move the focus of the measurement outside of the laboratory. So, you know, instead of it being another way to capture information within the clinical environment, now can we actually go out to the persons home, and can we have a HCP go to their home and capture that, or can we capture it in a primary care setting or in a physio setting.”—Participant 4, Male, Academic
“ I think I’d like to see more, almost more guidance or help, almost from the accelerometer developers in terms of data output and how the researcher can use it right.”—Participant 3, Female, Academic
“Keep doing it. As I said we are still in the discovery phase with all this and we can only when we have understood enough will we be able to use it and understand what we can do with it. So for me at the moment it is working well in the sense that as I said we are finding out things that we would not have known before but how to implement those is the next step. For me there is still a lot of opportunities out there.”—Participant 5, Female, Academic
“Well we are not the end users or consumers of this, that’s the patient but we are very close to the patient and then there are the other researchers and we can already see that that is a problem and that there is a disconnect. I mean I can already see with some of the conversations with my academic partners, but then with the algorithm they are so vast. I think it is the right thing we are doing. It’s not ideal as I said because we can’t predict the future and if we could that would be great.”—Participant 17, Male, Industry
“We’re movement scientists, and I think we should never lose sight of the fact that whilst again we can use laboratory based motion capture systems, and we can use inertial sensors to capture movement, they can create a whole new paradigm of how we measure movement and behaviour but ultimately we should never try and remove the human from the loop.”—Participant 4, Male, Academic
“I would say that….they are not a pancea. They are….[laughs], there is this trend that everything needs to be measured and that because we may have so much data, that this is useful and this is better. And for some research questions, a questionnaire or a simple question may be better and there are many important things related to health that an activity monitor wouldn’t measure such as the type of activity for instance. And, I think that it is important to remember that. That sometimes we tend to go for the more complex technology and sometimes you would just need to talk more with the patient and give less devices.”—Participant 10, Female, Academic

### Theme 1: the positives and negatives of using wearable devices in research

Researchers offered a balanced and pragmatic view regarding the positives and negatives of wearable sensor technology. For the most part there was a belief that the positives outweighed any negatives because of the need to progress this area of research further (detailed further within Theme 5).

Researchers were overwhelmingly in support of wearable sensor technology because of the insights it can offer ahead of traditional questionnaire-based outcomes. Furthermore, remote monitoring in particular has become an area of specific interest as it provides unprecedented access to objective, real-world data, and clinical insights into patient behaviours that were previously inaccessible. For the most part, researchers remarked that wearable devices are generally well accepted by study participants, who typically continue to comply even if some discomfort is present.

“*It’s what that data can tell us is the real positive. It’s not about the capture, it’s about what we can learn. I think we can learn so much more about people, you know, to help us manage them, to help us learn what works and what doesn’t work, to help us make them better sports people, to help them recover.*”—Participant 4, Male, Academic

This is still a rapidly evolving area and as such, there remains a number of problems with wearable sensor technology which researchers need to consider. Some issues relate to the study participant (e.g. skin irritation, device loss, incorrect attachments, not wishing to wear a device etc.) while others relate to how the researcher interacts with the wearable device (e.g. is it valid, no integration of multiple data streams or information about environmental factors). The design of a wearable device will impact study participant compliance. Thus, researchers have learned which wearable devices won’t be tolerated by study participants (e.g. ankle-worn devices) and how certain features are considered non-negotiable (e.g. raw data access, battery life of a week, validity). Furthermore, a certain level of competence is required by those using the wearable devices, both by the study participant and the researcher. Typically, when researchers consider these issues as part of wearable device selection, the biggest risk is that the negative issues will result in either data loss, or poor-quality data, which ultimately is what researchers wish to avoid (discussed further in Theme 4).

*“It’s not so much the burden of the eh, charging the device, it’s the fact that if the device isn’t charged then you lose those days…..if you’re only collecting seven days of data and you know you miss one day or something, you’re down 14% of data ... It’s like a big deal,….again it’s about the compliance and the continuity within the project.” -* Participant 6, Male, Academic

### Theme 2: the routine implementation of wearables into research and clinical practice

Although the positives of wearable sensor technology were clear from a research perspective, what was less clear to all involved, was how wearable devices may be routinely and easily implemented into practice. Regardless of whether researchers came from a clinical or technical background, the current iteration of wearable devices does not offer a clearly identifiable, easily understood, clinical utility.

There was agreement among researchers that this is an area at somewhat of a crossroads. Wearable devices are widely available from many avenues, and yet, it is not always clear how they are useful clinically. Researchers who are experienced with using wearable devices, and who can take the time to learn how to set them up, handle, and manage their data, can see their worth. However, that has not yet transferred into explicit clinical usefulness. This is partly because there is a perception that clinicians are not sure about what they want from these wearable devices, either because this type of technology has not been readily available to them, or because the technology cannot currently measure what would be useful, with one researcher remarking that they “cannot understand what they [clinicians] extract out of sensors that is clinically meaningful”.

“*For me, … it’s a bit more abstract, but we really need to think about how we are going to get people to use these tools in the clinic and how patients are going to use them in the home. What is it that we are going to do that will make it routine practice? What do we have to do to get there? And I think we’re still, there’s a lot of assumptions of course.… That’s still one of my motivating, one of my drivers because I believe that we can improve assessment, I believe we can improve patient feedback, but what is it that will actually get us to that point.*” - Participant 12, Female, Academic

Linked inherently to the uncertainty of how wearable devices, and their learnings, may be integrated into practice, is the role of the clinician in this process. Although they are positive about the potential that wearable devices may offer, there is a gap between what is currently possible and what may be promised by them. Typically, the depth of information provided by wearable devices that allow clinicians to access raw data, requires a level of data processing skills that clinicians often do not have, and do not routinely need for their role. While consumer-based devices are both easy to set up and use, typically, what can be learned from them may only be summary-level, basic insights. Furthermore, there were concerns around the validity of consumer-based devices. Nonetheless, some researchers spoke about the value that their study participants gained from these basic insights including increased motivation to wear the device. Therefore, it remains to be seen how this area will progress. Do the insights provided by the data captured from wearable devices outweigh their cost and burden? Can wearable sensor technologies provide meaningful clinical insights instead of simply generating data that does not augment the clinical decision-making process ? While this is still being established, multi-disciplinary teams offer the best method of combining insights from both clinical and technical worlds.

“*There’s no system that fits all the clinical needs. So the system that you use in the clinic is a completely different system than you’re using outside the clinic. And I think the biggest issue that I would say is with clinicians, because often clinicians don’t really understand the outcome of what you actually measure. So the whole data processing part is not very well understood*.”—Participant 1, Male, Academic

### Theme 3: the importance of compromise in protocols

Beyond the features and possibilities of the wearable devices themselves, it was obvious that their usefulness is only as robust as the protocols which are designed to implement them. However, pragmatism is needed as it is clear that what is considered ideal from a research perspective and what is practical, are often not the same. Frequently, what is desired from a technical perspective may not be acceptable from a clinical perspective. Furthermore, it is often a case of trial and error with protocols, as device specific learnings may only come to light in certain contexts. Ultimately however, although researchers have the ability to collect data in a range of ways, it doesn’t mean that they should. Any data collected should reflect the research question under investigation. Moreover, because of the range of wearable devices that are available, researchers noted that it was often a case of ‘better the devil you know’ in that they frequently chose devices they were familiar with and knew were valid. The result is that studies are limited in their ability to compare results because of a lack of standardisation and harmonisation between devices.

“*If you want good data, so if you need to do a proper gait analysis study, I would always go for pelvis. Well no, I would always go for two sensors on the ankles or on the foot. Not being able to do that, if you have to choose just one sensor and one location, I would always go for the pelvis. If it were wearability, so if I am more interested just in behaviour, I would probably go for the wrist*”—Participant 5, Female, Academic

“*If you want continuous monitoring you can’t wire them up like Christmas trees, just clinical tests or tests once a week at home and that might be possible*.”—Participant 17, Male, Industry

### Theme 4: Securing good quality data

The priority of most protocols is to balance the burden on study participants, while simultaneously avoiding data loss. However, a number of participant- and researcher-related challenges remain when it comes to collecting high quality data.

Study participants with chronic health conditions are often motivated to help improve the health of other patients who will come after them, irrespective of whether there is a direct benefit to themselves or not. Thus, these study participants are seen to be a willing and compliant cohort. Researchers were unclear as to whether they should provide study participants with performance-related feedback from wearable devices or not. The value of such feedback was highlighted in terms of motivating study participants and ensuring that they are comfortable that the device is working. Indeed, study participants’ interactions with wearable devices that they thought were broken, often inadvertently produced data loss, as they sought to fix the perceived problem. Therefore, it was noted that the risk of unwanted change in participants’ behaviour with limited feedback, was likely to be lower than the risk of data loss or reduced compliance as a result of no feedback. Nonetheless, ideas were provided as to how feedback could be provided without risking changes to behaviour, such as confirmation of data uploads or wear-time.

“*For some of the patients it would be good to have it be able to give them some feedback, as in a light, that they know that it is working or that it is doing something. Because they are like do I need to do anything? Do I need to turn it on? Do I need to charge it? And I think sometimes there is a comfort that they are not wearing it for no reason because they can see that it is doing something*.”—Participant 7, Female, Academic

Researchers noted the value of providing study participants with clear instructions on how the wearable device works, what data is collected by them, what is needed from them, how to put it on or take it off, and how to charge it. These are essential steps to support remote data collection. However, despite the acknowledged importance of planning for good data collection, researchers often do not undertake formal usability testing prior to implementing a wearable device. Instead, they talk to colleagues and undertake dry-runs using their own research teams in order to help determine what device suits their needs best. Associated with this is the need to conduct researcher training in how to run the protocols appropriately. This is particularly necessary when multiple sites are involved in various locations.

“*They [participants] might have questions so you need to give them some information before you attach the sensor and we have tried to not say too much about being an activity monitor but saying something like being a sensor that measures movement but it will not capture any pictures and so on. So you need to explain it and it is okay for most of them I think*.”–Participant 11, Female, Academic

*“ So in principle it’s the same as what I said about the patient. You know if you just tell them [collaborators at other sites] I have to do this and that and follow the protocol and then stick a wearable to the patient, then they may do it but they may not be very enthusiastic about it. If you involve them then that’s a different story then hopefully they’ll maybe transfer that to the patient*”—participant 17, Male, Industry

Although wearable sensor technology provides researchers with previously inaccessible information, a significant challenge remains regarding the inability to integrate multiple outputs and confusion surrounding how companies conduct their own analyses. Specifically, researchers frequently described the outputs from wearable devices as being like a ‘black box’. While access to raw data can support researchers in making sense of this output, they remarked that it was often still unclear exactly what wearable devices can measure. Furthermore, classifying activities remains problematic in populations with mobility issues such as slower walking or shuffling gaits.

“*We don't necessarily know what is happening in that black box of the analysis. They just give you the reports. We have got the raw data, we will probably be analysing it but for the most part it is probably here is a report, this the percentage. But I kind of want to check that*.”—Participant 7, Female, Academic

### Theme 5: a paradigm shift

Overwhelmingly, experienced researchers who have been working in this field for over a decade or more, consistently noted that they felt this area of research represents a paradigm shift in terms of understanding people’s movements. There was a clear feeling that, although wearable sensor technology does not currently provide the depth of insights that might be desired, that this is a rapidly changing area and one that has developed enormously in a short space of time. Consequently, researchers were pragmatic in their acceptance of some of the current challenges. For instance, they acknowledged that the promise of ‘simple’ behaviour change, whereby an individual simply puts on a wearable device and will change their behaviour based on its feedback, cannot be realised. Furthermore, they also recognise that problems and uncertainty are part of the process given the development stage this research finds itself in. As a result, wearable sensor technology is not currently the ‘game-changer’ that it was originally purported to be.

“*It is a technical development which may be relevant for learning more about human behaviour within the next decade or two and then I am convinced that the new technology will substitute this technology. This is how it always happens during evolution and even scientific evolution, now we are in a phase where we can collect data with such types of technology*.”—Participant 16, Male, Academic

“*I think there’s a kind of fundamental belief that this is the way that we will measure people in the future. It offers us … a completely new paradigm for trying to understand human behaviour and understand how that changes with different clinical conditions, and how it can change with different treatment interventions*.”—Participant 4, Male, Academic

Nonetheless, researchers highlighted the areas that they felt needed to change, or could be improved on, in order to further progress this paradigm shift. Specifically, there was acknowledgement that researchers must continue to collect large datasets, in a variety of cohorts, using a variety of devices and a variety of attachment sites, in order to understand the context of movement, or behaviour, and create digital biomarkers of health. It is only through gathering and understanding this data, that the area will be able to produce standards for what works best for what research question. Furthermore, multi-disciplinary teams were considered vital in the development of wearables. Clinicians, academics, engineers and wearable manufacturers need to continue to build their relationships and interact in order to best understand the needs of all stakeholders in all aspects of wearable device design and implementation. There was no firm consensus regarding what specifically needs to change, reflecting the complexity of the area, the research priorities of various patient populations, and the different experiences of researchers in this area. Nonetheless, a number of ideas were provided, some of which focused on the design of the wearable devices, and some which sought to plot an ‘ideal’ pathway in which the research could be undertaken. Examples included producing smaller devices, integrating devices into shoes and clothing more, being able to capture contextual factors of movements within a wearable device, measuring people only as much as you need to, and developing manufacturing standards that will allow an integration of data streams or ‘bring your own device’ studies in the future. Within this, however, one researcher remarked that despite all the improvements in this area, and the future capabilities of wearable sensor technology, it will remain necessary to ‘keep the human in the loop’ in order to best understand wearable device outputs and to derive clinical meaning from them. Ultimately, for all its promise, the advance of technology cannot come without the expertise of the people driving it.

“*And if you had a standard, theoretically if a company was producing you know, if they were measuring acceleration and gyro, and it’s on the same part of the body, now going from wrist to waist now is probably a really difficult one but you know if there’s a standard around the sensor characteristics or the measurement capabilities, then theoretically you should be able to go from manufacturer to manufacturer and learn the same things*.”—Participant 4, Male, Academic

“*I just think it’s really important for us to get the conceptual framework of what you’re measuring, what does it mean, and you know what interventions are available. So it’s going to be different for different devices, monitoring outputs, different populations*.”—Participant 19, Male, Academic

## Discussion

This qualitative study explored the experiences and opinions of researchers from academic, industry and clinical contexts, in the use of wearable sensor technology to collect gait and physical activity data. The results provide insights into where this area of research is going and what needs to change to allow it to deliver its promise of revolutionising healthcare. Specifically, although the benefits of wearable devices are well known, it is not yet clear how they can be integrated routinely into practice. This is because analysing raw data is a specific skill in itself that is not easily undertaken by those in clinical contexts, while integrating and comparing the data streams from multiple wearable devices is not yet possible. Essentially, the use of wearable sensor technology represents a paradigm shift in how we understand human behaviour, and as with any shift, significant learnings and process changes are needed to maximise the capability of these devices.

Participants shared the beliefs of others that the data gained from wearables will help the management of various chronic conditions [1, 3]. However, identified challenges were also similar to that of previous research focusing on patient and clinician opinions [1, 2, 11, 15, 17, 18, 24]. Data management and analysis in particular was listed as a major problem irrespective of the background of the researcher. Essentially, researchers do not trust ‘black box’ processing from any wearable device and prefer to either validate it themselves or have access to its raw data. This is unsurprising given that study results rely on reliable, accurate data, and indeed this has been the focus of much of the research in this area up until now [25]. The need for digital solutions to be adopted and scaled into health services is considered a key objective for this area going forward [3]. However, the reliance on raw data brings with it the need for a certain level of competency in data management, a skill which remains daunting to those from clinical backgrounds [8]. It has been suggested that the creation of perfect research conditions limits clinical transference of protocols [26] and it seems that this is still a barrier for many researchers in this area. While multi-disciplinary research teams are used to balance the technical and clinical needs of a project, until data handling becomes manageable by all, regardless of their background, wearable devices risk remaining as ‘nice to have’ rather than ‘need to have’ products.

The relative modernity of these devices means that they are often tested in simulated or highly-controlled environments, thus, learnings from remote contexts are only recently emerging [8]. For the most part, these are gained through trial and error or through the use of ‘friendly’ in-house trials. However, it was notable how few researchers conduct formal usability evaluations as part of their data collection protocols. Measuring the attitudes to, and engagement with, digital health is considered to be an integral part of its progression, and the WHO is working closely with the ISO to ensure that person-centred care is a focus of any developments [3]. Thus, the finding that this concept is not formally evaluated by many in this area of research is disappointing. Researchers are strongly advised to document their experiences and learnings in a more structured manner in the future. The use of fidelity guidelines may help to ensure that they maximise the efficiency and cost of their trials by designing protocols, training staff, and evaluating the delivery of a protocol from the perspective of both researchers and study participants [27]. Combined, these would offer researchers a more robust method of capturing barriers to wearable sensor technology and their procedures, thus allowing them to formally compare multiple wearable devices across various cohorts. Furthermore, without this, researchers are currently relying on using the same wearable devices because they know their faults and won’t be surprised by them. The cost of new devices and testing their validity is also a potential barrier to trying different systems, and so researchers felt it was ‘easier’ and more cost-effective to not stray too far from the familiar. However, the result of this is that device progress is potentially being restricted as researchers are not readily trialling new devices for fear of what they may lose because of it.

To the author’s knowledge, this is the first study to explore the experiences of researchers in their use of wearable sensor technology. Valuable insights have been gained into understanding the challenges that continue to exist in this critical area of research, from both a technical and clinical perspective. It is clear that we are far from the original idea that wearable devices would simply need to be deployed to change healthcare [4]. However, critically, this was not seen as a negative. Rather, there was acceptance that wearable sensor technology, the data it produces, and implementing them, is far more complex than first envisaged. Complex problems are often said to require complex solutions, and this seems to be the case with wearable devices. Nonetheless, the complexity almost adds to their potential, in that researchers who have witnessed their progression, are passionate and convinced that this will be the game changer that was planned, but simply acknowledge that it will take longer than initially intended. Indeed, one of the most important findings was the certainty in which researchers spoke about this being a paradigm shift in which research attempts to not only better understand human movement and behaviours, but understand how it can be best captured and, most importantly, what can subsequently be done with this information.

However, this study is limited by its use of researchers from a single consortium and its associated focus on wearable devices that measure gait and physical activity. Specifically, it is likely that the researchers from this consortium, as a result of their work in this area, are positively biased towards the use of wearable devices. Nonetheless, the Mobilise-D consortium is multi-disciplinary in nature and the participants in this study represent a broad mix of backgrounds and experiences across multiple sites in nine different countries across Europe. Therefore, it is likely that the experiences of these researchers are representative of the field overall. However, it is worth noting that many of the researchers expressed a bias against the use of consumer-based devices for reasons of validity. Additionally, researchers were not followed up with additional interviews or collective discussion in focus groups, which may have provided further insights into specific findings such as the direction and changes needed for research in this area. Finally, two of the three coders used to analyse the data were from physiotherapy backgrounds, therefore it is possible that a classification bias may exist in how codes were generated.

Participants differed in their opinions regarding the next steps for this area of research. However, all agreed this field will not improve without a multi-disciplinary approach, and most critically, an ability to compare data sets [3]. This requires significant work from, and between, wearable device manufacturers regarding the availability of their processing algorithms [3, 28]. Rapid, scalable processing solutions that work across multiple wearable devices are also required [3, 8], while platforms need to communicate between each other to break down the barriers and uncertainty regarding data management [3, 28]. Academically, multi-disciplinary work that collaborates with industry, patients, researchers, and clinicians is required to ensure that the needs of all stakeholders are met. Indeed, projects such as Mobilise-D [20] have recognised this and are implementing and optimising existing algorithms in real-world data. However, these need to become the norm and not the exception if the promise of the paradigm shift is to be realised over the coming years.

## Conclusions

Significant changes have emerged in the measurement of human movement in recent years with the evolution of wearable sensor technology. This development is the result of substantial work from academics, clinicians and industry, the outcome of which is a new paradigm for how research in this area is conducted. However, despite the advances, multiple barriers to the use of wearable devices in research and clinical practice remain. In particular the following barriers are worth noting:

- A clear, clinical utility for the output derived from wearable sensor technology has not yet been identified and implemented into practice.
- The management of data derived from wearable sensor technology still requires a set of skills that many clinicians do not have. The result of this is a continued reliance on proprietary algorithms or a need for multi-disciplinary teams to handle data processing and analysis.
- Capturing good quality data in the ‘home’ environment of any clinical cohort is a complex matter that requires clear planning, well-defined protocols that consider the challenges and barriers of remote monitoring (i.e., interactions with daily living, battery life, patient abilities, clinical versus technical needs).

Nonetheless, researchers strongly believe that the potential benefit of wearable sensor technology to support and create new clinical insights for patient care, is greater than any current barrier. Multi-disciplinary, publicly available research integrating the expertise of both academia, healthcare and industry is a fundamental necessity to further develop wearable devices and protocols that match the varied needs of all stakeholders. Future research utilising wearable sensor technology to capture gait and physical activity is advised to consider the following:

- Conduct structured and formal usability assessments as standard when implementing wearable sensor technology.
- Continue to capture rich data, at scale, in multiple cohorts and with a variety of devices in order to increase our understanding of human behaviours, including how it can be best captured and what can be learned from this information.
- Conduct collaborations between academic, clinical and industry-based partners in order to maximise the learnings and expertise from each group and to build protocols that consider the needs of all stakeholders.

## Supplementary Information

**Additional file 1.** Expert learnings regarding the use of wearables in healthcare research to measure PA in older adults.

**Additional file 2.** Expert interviews for Mobilise-D—Coding book.

## Data Availability

The datasets generated and/or analysed during the current study are not publicly available due to the potential for identifying researchers through their responses within the full transcripts but are available from the corresponding author on reasonable request.

## Abbreviations

ISO: International Organization for Standardization
WHO: World Health Organisation
