# Supplementary material for: It’s not about the capture, it’s about what we can learn”: a qualitative study of experts’ opinions and experiences regarding the use of wearable sensors to measure gait and physical activity
Source: J Neuroeng Rehabil. 2021 May 11;18:78. doi: 10.1186/s12984-021-00874-8 (PMC8111746; doi:10.1186/s12984-021-00874-8)
Supplement: Supplementary file 1 — Additional file 1. Expert learnings regarding the use of wearables in healthcare research to measure PA in older adults. [file 12984_2021_874_MOESM1_ESM.docx]

**Expert learnings regarding the use of wearables in healthcare research to measure PA in older adults**

**Background:** We often examine usability and human factors from the perspective of the participant. However, there is a wealth of potential learnings and experiences that exist amongst the researchers and academics who implement them. Specifically, it is important to understand how researchers view the use of wearables in healthcare, their perceived barriers and facilitators, and their perceived barriers and facilitators in relation to the learnings that they have gained from participants, and the usability factors that researchers perceive to be the most important or influential when selecting a device.

**Aim:** To explore the experiences of researchers in the use of wearable devices to measure PA in older adults.

**Included questions**

**Open: aim of interview, amount of time, any questions?**

1. **Can you tell me what your experiences have been with wearable devices to measure PA to date?**

Potential prompts (use if required):

- 1. Positive?
     1. What do you like?
     2. What are the benefits?
     3. What about from the participants?
  2. Negative?
     1. What do you not like?
     2. What are the downsides?
     3. What about from the participants?
  3. Experiences of researcher in terms of years with sensors, whether they interacted with them themselves or via others, number of sensors used etc.
     1. What sort of devices have you used?
  4. What is your opinion on the use of wearable devices and technology in the assessment of PA?

1. **How do you select a device for a study or clinical trial?**

Potential prompts (use if required):

- 1. What aspects do you prioritise?
     1. Technical?
     2. Usability?
     3. Participant v’s researcher?
     4. Support?
  2. What would make you stop using a device, or stop you from choosing to use it again?
     1. Technical?
     2. Usability?
     3. Participant v’s researcher?
     4. Support?
  3. What have been the devices that you prioritise using and why?
     1. Would this change depending on the study setting (i.e. home v’s lab)

1. **From your experiences with using various devices, what are your opinions as to what participants think of these devices?**

Potential prompts (use if required):

- 1. What does the term usability mean to you when we talk about wearable devices?
     1. And what about for the participant?
     2. And what about human factors?
  2. How much do usability features influence your decision?
     1. Which features?
     2. How do they assess usability?
     3. How is your choice of device influenced by the environment that you will be testing in?
  3. What about the human factors?
     1. When it comes to participant experiences with wearable devices, what have you learned from participants in terms of how they feel about using wearable devices?
     2. Required interaction
     3. Comfort
     4. Location
     5. Users expectations of the device v’s reality

**Ends interview with round up and any questions?**

**Role**

**Years with wearables**

**Years in research**

**Qualification**
