# Supplementary material for: It’s not about the capture, it’s about what we can learn”: a qualitative study of experts’ opinions and experiences regarding the use of wearable sensors to measure gait and physical activity
Source: J Neuroeng Rehabil. 2021 May 11;18:78. doi: 10.1186/s12984-021-00874-8 (PMC8111746; doi:10.1186/s12984-021-00874-8)
Supplement: Supplementary file 2 — Additional file 2. Expert interviews for Mobilise-D—Coding book. [file 12984_2021_874_MOESM2_ESM.docx]

**Expert interviews for Mobilise-D – Coding book**

Background

We often examine usability and human factors (understandably), from the perspective of the participant. However, there is a wealth of potential learnings and experiences that exist amongst the researchers and academics who implement them. Specifically, it is important to understand how researchers view the use of wearables in healthcare, their perceived barriers and facilitators, and their perceived barriers and facilitators in relation to the learnings that they have gained from participants, and the usability factors that researchers perceive to be the most important or influential when selecting a device.

**Aim**

To explore the experiences of researchers in the use of wearable devices to measure PA in older adults.

## Coding methods

**Assumptions and biases**

This study took place as part of a specific work package task to identify the human factors and usability issues associated with the use of wearables for the remote monitoring of gait and mobility. Therefore, prior to conducting this study, participants were aware of the proposed study and its purposes, and some may have already engaged in discussions with AK and other consortium members around the topic of usability. Thus, a professional relationship already existed between AK and some of the interview participants prior to conducting the interviews.

All interviews were conducted by AK, a female physiotherapist with a PhD in behaviour change and a post-doctoral researcher in wearable technology, who had previously undertaken qualitative research exploring the concept of usability with wearable devices. Therefore, AK entered the interviews with previous knowledge as to some of the common barriers and facilitators behind the devices, from a clinical perspective. AK is also a member of the Mobilise-D consortium, and therefore was part of the same collaborative consortium as all participants within this study. Interviews will be coded by three researchers, AK, BV and KT. BV is a female Professor of Movement Science. KT is a female physiotherapist with a PhD in Clinical Medicine. Both BV and KT are also members of the Mobilise-D consortium.

The interviews will be coded using an inductive thematic analysis from a realist perspective, whereby it is assumed that the interviews report the experiences, meanings and reality of the participants (Braun and Clarke, 2006).

When developing the interview topic guide, the Technology Acceptance Model (TAM) was used a theoretical guide (Lee et al., 2003). This posits that technology is adopted when it is easy use and is perceived to be useful to participants. Questions therefore related to participants experiences of wearables, including the barriers and facilitators of their use so as to explore the assumptions of TAM. However, the interviews will be analysed using an inductive approach whereby codes and themes will be identified based on the data itself, rather than using any specific theoretical constructs or coding framework (Braun and Clarke, 2006). A rich thematic description will be sought so that predominant and important themes throughout the full dataset were identified, rather than searching for detail on a specific topic.

**Coding process**

The coding process follows that outlined and suggested in previous research (Braun and Clarke, 2006). The interviewer (AK) will familiarise themselves with the full data set initially by reading all transcripts once, noting any thoughts that arose within a coding notebook. From there, all interviews will be read a second time, with initial codes subscribed to each interview.

An initial coding book will then be developed and sent to two other raters for review (BV and KT). KT and AK coded one transcript each, at which point their agreement was assessed and discrepancies in coding were noted. Changes to the coding book were made and agreed upon by AK and KT. The next draft of the book was used by KT, BV and AK to code another transcript.

A meeting was then be held between the three raters to discuss any discrepancies, to identify any additional codes and to finalise the coding book. From there, each of the texts will be coded for a final time by AK (n=20) using this coding book. A further three texts each will coded by BV and KT so that in total 50% (n=10) of texts will be double rated. Inter-rater reliability will then be established using percentage agreement).

**Identification of themes**

Once coding is complete, codes will be collated into potential themes and subthemes where patterns are identified by AK, BV and KT. A thematic map will be created and these themes and subthemes will subsequently be refined through discussion with all three raters. Compelling extracts from the interviews will be identified by each rater to ensure that the themes represent the data set before a final set of themes will be agreed upon.

## **Coding instructions**

## In a piece of text, try to only code the primary code that it relates to, rather than multiple codes. However, if you are unsure as to which code suits the passage of text best, then please code both and include a comment on what you think.

In a paragraph, please code each sentence individually with the codes that you identify.

Finally, once you finished coding a text, please comment on your overall impressions and learnings from the interview.

## Coding book

The Table below represents the third draft of the coding book, as discussed between AK, KT and BV. The Table outlines the name of the code, it’s definition in relation to this study, and examples of what it does and does not include.

Please review the text sent to you for the presence of these codes. Some codes may not be present in all texts. In addition, some areas of text may relate to multiple codes. **If while reviewing a text you feel that other codes exist but are not represented in this Table, please note these and where you see them and label them as “Other’.** Additionally, if you feel that some codes within this Table are not relevant to the dataset or are not required please highlight them and suggest reasons as to why. There may be some overlap between certain codes. We can discuss whether we may be able to merge codes, or whether this reflects a particular pattern. Finally, if any codes are particularly difficult to identify or you are uncertain about them, please also note these codes for discussion.

Please use the comments function in Microsoft Word to code areas of text.

**Finally, it would be useful for each rater to provide an overview of what they found in each text, or what they feel the main messages were in each texts. This only needs to be short (3-4 lines). It may be useful for us to compare this once all transcripts are coded. It may also help us to identify the overall themes.**

Twenty-three codes have been identified and are listed alphabetically:

1. Barriers and challenges to wearable device use in research
2. Benefits of using wearables in research
3. Conflicting needs
4. Consumer/Commercial v’s Medical device
5. Contextual information about the researcher
6. Competencies
7. Developments in wearables since conception
8. Device requirements
9. Device specific learnings from research studies
10. Expectations v’s Reality
11. Feedback and information from the device
12. How the research aim and design influences wearable device selection
13. Impact of device on patients’ lives in a manner that influences the outcome derived from the sensor
14. Participants opinions regarding acceptability of devices
15. Personal experience of researchers wearing sensors
16. Privacy
17. Research design challenges
18. Reasons why people participate in research
19. Remote monitoring- the impact of context on research
20. Strategies to enhance adherence to the study protocols
21. The future of wearables
22. Usability testing
23. Other

|  | **Code name** | **Code definition** | **Example** |
| --- | --- | --- | --- |
| **1** | **Barriers and challenges to wearable device use in research** | Challenges that researchers have encountered with wearables in their studies.  Includes:   - Design - Functionality - Cost - Participant interaction with the device - Battery length - Device size - Method of attachment - Problems with data upload or management - Validity of device   In essence, this refers to device-related issues that can negatively impact a research study.  **NOTE:** This can be differentiated from ‘device requirements’ if the researcher suggests that it is something that they have ***previously*** experienced or learned, whereas ‘device requirements’ related to what they need to consider ***for future or current*** device selection.  This may also be coded as *‘Participants opinions regarding acceptability’* if there is evidence that the challenge has been described by the user. | *“Eh, another thing was we had a few times where essentially the accelerometer stopped working, meaning that the green light stopped flashing and so it was a bit of a hassle both for us and also the participant to switch it as quickly as possible. So essentially what ended up happening is that we started giving the participants two accelerometers so that one could be replaced if that were to happen but ultimately meant at our end that we were down a number of accelerometers because we had to cut our number in half”*  *“A lot of times we want to use the sensors and use several sensors simultaneously so you need to keep track of which sensor you are using, which is the sim card, for the right or the left, or the waist or the chest so you need to keep track on that. Eh, and as I said also the battery so you need to keep track that the sensors are properly charged, that they are uploaded, that they are prepared to record the data, that they don’t have data from previous measurements”*  “*I am not that software engineer so sometimes you upload the data directly to their service and from a clinical point of view you then look into the reports as soon as they are processed. Also because we are not that proficient with the scripting and the algorithms we tried to stay away from that as well, because you don't have enough background, you don't trust yourself fully enough*.” |
| **2** | **Benefits of using wearables in research** | Researchers opinions as to the general benefits of using wearables.  Benefits can be related to:   - Healthcare - Research practices - Global understanding about conditions - Reasons as to why they use wearables in research.   **NOTE:** This should be differentiated from ‘*Participants opinions regarding acceptability*’ where possible, according to whether the researcher says it or whether they refer to what the participant said. If the researcher states that the participant reported it, then code as ‘participants opinion regarding acceptability’. Otherwise, code as ‘benefits of using wearables in research’. | *“So obviously the activity monitors allow us to obtain some information that with other measures, with questionnaires, would be biased with definitions so this systematic error of some people reporting more and other people reporting less. They provide you with this information that you cannot obtain otherwise.”*  *“we do not know a lot about certain diagnosis and how they perform in a way in the home environment. We know as clinicians a lot about how people perform in the hospital and the doctor's practice but we do not know what they do in the home environment. So we are not aware of many symptoms that may exist and do exist but we have never seen them, we have never understood them as medical professionals”* |
| **3** | **Conflicting needs** | Any sort of example or incident whereby researchers must consider the needs of one variable over another, or where they need to compromise because of competing interests or need.  Examples include:   - The needs of participants v research needs in terms of device selection - The clinician needs v technical abilities when choosing a device - The ideas of clinical v technical research staff in terms of the research protocol or study design - Participant preferences and data capture abilities in terms of which device to select - There must be evidence of compromise or favouring one factor/stakeholder group over another   **NOTE:** This does not include examples of where a researcher identifies that they do not need additional skills or input because of the wearable. | “*So, I, the sample size is based on the research question and the characteristics of the main outcome measure, so its fixed. Then, depending on the research question and the population I select the best accelerometer. And then if, for instance this is more expensive, I would extend the agreement period so that I don’t need to buy 10 or 50, and I buy 10 and I have a longer agreement period. That would be my margin.”*  *“So I think from a research perspective I don’t have a problem when I have to program the device for 10 minutes or 20 minutes and then I know that the patient has not to do anything just wear it then it’s definitely a trade that I would do because otherwise you end up with a system that’s maybe very easy to start, you just hit start here and you give it to the patient but they have to charge it every night, and call you and tell you that you’re a moron because that’s a shitty device*” |
| **4** | **Consumer v Medical devices** | Anything that compares the differences between consumer grade and medical grade devices.   - Consumer grade devices in this case may also be called commercial devices. - Refers to devices which are for sale to the general population (e.g. Fitbit). - Includes any reason why a researcher would or wouldn’t choose one or the other | *“I was using pedometers that we brought at Decathalon so they were the cheapest ones and that was quite useful for feedback. So as I said it depends on the research question, if you want to use this to measure I would never use one of these but if it’s for feedback for patients then that’s ok because that’s simple and it’s very cheap and I have used also some Polar ones, pedometers, for feedback purposes”*  *“Sometimes you are aiming for high validity and other times you may be more interested in high sensitivity to change. So then you choose one thing or another.”* |
| **5** | **Contextual information on the researcher** | When the researcher provides information about the type of wearables that they have used before, without providing any opinion on these devices, or what they learned from them. |  |
| **6** | **Competencies** | The skills needed by anyone involved in the implementation of the study protocol to accurately use wearables in research.    If a lack of skills is listed and it influences research design then it should be included.  Includes:   - Clinicians - Lab technicians - Upskilling by research assistants etc. - Examples where a researcher suggests that little to no skill is required for device use. | *“See I think the issue is that, I don’t, like I don’t understand the software inside the accelerometer. Like I don’t even fully grasp the potential it has. Right? Maybe there are just on the software developer side a few things that need to be changed and then I actually get the perfect output that I need but I’m not even aware of this because I’m not like, I don’t have that kind of training to even be aware of it.”*  *“I say it’s easy to set them up but of course you need to have some sort of experience on that otherwise you can run into mistakes. Like maybe removing the data by accident or using the SIM card that you’re supposed to be using but you didn’t and confusing the participant, or typical mistakes yeah when you measure.”* |
| **7** | **Developments in wearables since conception** | Researchers accounts or opinions of the changes or progressions in this area of research over time.   - This relates to the ‘big picture’ of wearables - Where they fit in the research paradigm. - Relates to changes seen in wearables overall, rather than specific devices.   **NOTE: Do not code this if** the research speaks about recent changes to a specific device. If they do this, consider coding ‘device specific learnings from studies instead’ | *“I think that there has been a massive, I mean for me it has been interesting to observe in the sense that coming from the technical background, seeing the uptake before these products were actually ready to be implemented the most interesting part of the story. And somehow it is something that reminded me of what happened with motion capture back in the days. Everyone started having these labs where they think that it was just something that you started using”* |
| **8** | **Device requirements** | Anything that a researcher thinks about or considers when they are selecting a device in a study.  Examples include:   - Participant factors - Technical requirements - Cost - Comfort - Aesthetics - Method of attachment - Anything that may support participants to use wearable devices - Anything researchers have identified as being important to participants in order to support their use of a device   **NOTE:** This can be differentiated from ‘barriers and challenges’ if the researcher suggests that it is something that they need ***to consider for future or current device selection***, whereas ‘barriers and challenges’ relate to something that they have experienced previously. | “*I’m coming from the physical activity angle so two things that are key for me are weight, so weight and size of the device. That won’t really change, that’s just always going to be important. I think the other one would be battery capacity, then the next one would be water proofness*,”  “*I think form factor is really important because it has to be comfortable. Eh, if we look at the usability first the form factor is really important. The ease of putting it on and taking it off, we’ve worked with some sensors where the straps are just awful and they were just not easy to put on and take off. The social element of it. You know I’m generally not a fan of anything that goes around the waist because you’re thinking well would I like to wear that if I was at work and I had to have a suit on because it doesn’t look like it’s that easy to fit under a suit. I’m certainly not wanting to put it over a shirt! Eh the charge time or the battery life should I say is so important. Being able to, because that’s part of, you know ultimately it’s the deploy and forget, that’s the critical thing you know what are the different things that come into deploy and forget.”*  *“Another thing is if it provides accelerometers and gyroscropes or only accelerometers, and life battery. So if the life battery was not enough long in order to include gyroscopes or not so it was a trade off, you have gyroscope normally the battery of recording is shorter.”* |
| **9** | **Device specific learnings from research studies** | Device specific information and learnings based on researchers using wearables in their own research. This relates to specific details or opinions about named devices. | “Axivity because it was the smallest, one of the easiest to set up and due to the size you can either put it as an additive or you can attach it if you like to small belt or so I would say size, length of battery and price, this was the best.” |
| **10** | **Expectations v Reality** | Comments relating to what any stakeholder or user expects from wearables that may be different to what is possible.  Examples include:   - Patient expectations - Clinician expectations - Research collaborator expectations | *“I think the expectations are often completely different between when you compare clinicians and researchers, or technicians. So what is actually feasible to measure. So I think that is a big issue at the beginning but then throughout the experiment what often happens is that people want to change the protocol or they say, hey wouldn’t it be better if we put the sensor somewhere else, and you’re pretty much messed up*.” |
| **11** | **Feedback and information from the device** | Any opinion, positive or negative, from the researcher, in relation to feedback that comes from wearables.  Includes:   - What researchers think about whether they should provide performance or outcome based feedback to participants as part of the research study. - Reasons why they don’t give feedback   **Code only if:**   - The feedback has derived from the device itself based on past events or performances - The feedback must specifically be for the participant. - It can be feedback that comes directly from the device in real-time (e.g. step count) - It can be performance based feedback that has been generated from their data by the device software, or by the researchers themselves. | “*We don’t have devices that feedback in real time so its at the end and I don’t believe that there is a robust tool out there that allows people to have feedback in real time and that’s why we don’t use it.”*  *“I think that either so after a test or at the end of the day there should be a short summary saying eh, you have worn the device for however many hours and thank you very much. That’s sufficient for the overall monitoring and for the walk tests I would say thank you for conducting the walk test, we have estimates from this test that you have done 55 steps, your data has been uploaded online by someone, so just a few words that is has worked. The patient doesn’t even know whether it has worked or not and the patient may get frustrated if eh he doesn’t get any feedback.”* |
| **12** | **How the research aim and design influences wearable device selection** | Any reference to how the aim of the project/study influences device selection.  May include:   - Differences in the research design and considerations between lab-based or remote monitoring studies, - Differences in studies that monitor people remotely compared to studies which aim to change their behaviour. The sensor requirements for each setting.   **NOTE:** There may be overlap between this and ‘device requirements’. It is ok to code both at the same time. | *“Eh, so obviously the pattern of physical activity is different so if you have a slowly moving population you may be interested in a kind of device different than if you targeted healthy sports men. So things like that. Also then if we have children we may want for a not so expensive activity monitor because they may break it”*  *“No. So, I, the sample size is based on the research question and the characteristics of the main outcome measure, so its fixed. Then, depending on the research question and the population I select the best accelerometer.”*  *AK: So what is the difference for you in terms of sensor selection then for the two environments?*  *S1: I don't know when it is in the clinics I use multiple sensors normally in the sense that, I mean the same brand but multiple sensor locations to get more accurate data.*  *AK: And participants are okay with wearing multiple sensors at once?*  *S1: Yes because you don't have to get undressed, you don't have to... It is easy, if you put two or three or four it doesn't really change.*  *AK: And typically how long would those sessions be?*  *S1: It depends if they do a six minute work test which is what we use for most of the patients then it is that plus five to ten minutes to take the sensor on and off.*  *AK: For your home monitoring, is it typically a week, less, more? S1: Usually a week*.” |
| **13** | **Impact of device on patients’ lives in a manner that influences the outcome derived from the sensor** | Any reference to the how wearing a device may impact a person’s daily activities in a manner that will also influence the outcome of the study.  May be:   - Either in a positive or negative manner. - How the concept of burden influences research design or study protocols.   Examples include:   - Participant doesn’t walk as much because of the device (thus influencing outcome) - Participant needs to charge device frequently and may forget (thus leading to data loss)   **NOTE:** This should only relate to changes in the participants activity or behaviour that occurs as a result of the wearable, which may influence the study outcome. For example, if a device is simply prone to breaking or being lost, then this should be coded as a ‘*barrier or challenge’*. | “*Most of the time people who participate in studies are enthusiastic. They are willing to contribute and of course it is up to us not to over burden them or you will have failure. If you want continuous monitoring you can’t wire them up like Christmas trees, just clinical tests or tests once a week at home and that might be possible.”*  *“You come across the, sometimes it’s usually an engineer for example who has this thing of oh we’ll just ask them to do x and we’ll ask them to do y and we’ll ask them to plug this thing in here and that thing over there and sure it’ll be fine sure if I can figure it out they can figure it out. And then in the other extreme you have people say Oh my God we can’t ask them to do anything because it’s an awful burden. And the truth is somewhere in between”* |
| **14** | **Participants opinions regarding acceptability** | Researchers recall of what participants feel about wearing devices.  May include:   - Evidence that certain things are acceptable to participants (e.g. location, monitoring period, number of devices etc.) This can come as a result of specific usability testing, or as a result of researchers informal interactions with participants. The participants opinion may be positive or negative.   **NOTE:** For this to be coded, there must be evidence that the opinion given has come from the participants themselves, rather than simply being the researchers opinion.  May also be coded with ‘*device challenges’* especially if opinions are negative. | “*for example we had a focus group say that they didn’t like to have anything on their ankles because they felt it was tacked like for example people in prison*.”  “*We had feedback of people getting annoyed with the charging. Some of the charging hardware been used for a study wasn’t ideal, you know that tiny little USB cable that people were not able to fit in or charging stations that were loose and might not fit in properly and are falling out. All of this frustrates the patient and then the device falling off the foot for example was a problem. We even had reports of someone getting a skin rash from the device so the rubber or whatever caused the problem but of course then the patient will not then continue using that device*” |
| **15** | **Personal experience of researchers wearing sensors** | Researchers own experience of wearing devices and their learnings from this. In particular how researchers own wearing of a device may influence their device selection in a study. | “*The one thing I should add, from a research participant point of view, I participated in a study where I was asked to wear an accelerometer for a week but it was more like a, actually like a hip strap thing, where I had a little it looked like an old school camera where people would wear on a hip strap. And what I realised then was it was a real pain and it really limited me in my physical activities, the bouncing around*” |
| **16** | **Privacy** | Any reference to privacy issues either in relation to participants or researchers, or any reference as to how privacy may influence device selection.  Privacy in this case may refer to:   - What users believe happens to their data - How they are being monitored. - Specific questions or queries they have regarding who has access to their data - Questions about what happens to data as a result of using the wearable device. - Questions or concerns about being observed | *“I think people really need to understand what it does and what it doesn’t do. As I said it can be a privacy issue people saying does it track what I do, does it record what I say, and it’s important to have some instruction or be very clear on what they do.”*  *“patients here want to feel secure and they are very worried about their privacy so devices which record their I don’t know, cameras or voices, and this is something we’ve experience with, again it needs to be very clear to them why we are doing that and we need to really prove that it helps.”* |
| **17** | **Research design challenges** | Practical barriers or considerations to using wearables that are unrelated to either the participants or the device itself.  Examples include:   - Agreements with industry partners - The weather of the country they are in - The time of year the study takes place in   **NOTE:** **Do not code if** the challenge relates directly to the device itself. It is unlikely that this will be coded alongside ‘device challenges’ as the two are independent. | “*And also from the professionals perspective as well, how easy is it because you might be dealing with research nurses who are technophobes and we’re asking them to plug a device in, open up a computer programme, download the data, that is not simple. You know you’re asking about what have you learned, well we have in a big national cohort study that we’re involved in and where again we’re the team lead for wearables. In the feasibility study we had to go in and train the clinical sites to collect this data so we were training clinical researchers, associates or research nurses and we did learn a lot in that*.”  *“in our industry world you have a very complex, onboarding process of dealing with a vendor. Its complex, a lot of paperwork, a lot of due diligence and once you have that in, eh, you want to continue with that relationship so you are a bit forced to stay there”* |
| **18** | **Reasons why people participate in research** | Reasons why people participate in research.  This may include:   - Differences that exist between groups of participants in terms of how they participate in research or why - Cultural elements such as the country they reside in - Gender - Specific illnesses and conditions | Culture may be referred to by researchers who explain recruitment or participation based on the country someone is from, or the age profile of the participant.  “*But I think culture is important also. In different countries we do not only have different geographic conditions or climate conditions, we also have different behaviours. And different attitudes towards the health professionals and also different willingness to behave in or to participate in the group*.” |
| **19** | **Remote monitoring- the impact of context on research** | This refers to the challenges that are encountered because of real-life, contextual scenarios, or factors that relate to people’s lives, that only become clear after wearables are implemented.  May include:   - How context needs to be considered in study design - How context is difficult to capture and understand - How context needs to be accounted for in research design   **NOTE:** This relates to information that could have been known, until it was implemented. For example, if a device is difficult to use for people with problems with fine motor skills, then this is a contextual learning. It is not considered a problem with the device, as the device would be ok in another patient cohort. | “*Participants are usually pretty good at following the protocol you know so there is no issue with that. But it’s just the sort of real-world living conditions that you can’t account for in the lab like. If somebody goes out to the shops and they get soaking wet in the rain, they come home and they take everything off including your device and they don’t put it back on until their clothes are dry, you know. It’s that kind of stuff is the only real difference. The friendly trials for us are really just a confirmation that what we’re doing is you know, is good, has sound logic behind it and in practice it could work. But for the most part when we do friendly trials its healthy volunteers, so they don’t have the same needs and they don’t have the same concerns or worries that an individual might have in their home or outside of their home*.”  “*if you monitor someone for let’s say 12 hours a day over a longer period you get a lot of data which can be sort of perceived that you also get an insider knowledge of the patient and that might not be the case. So the lack of context is a problem so you don’t know where the patient is, if it’s just one device with no GPS, no nothing, are they outside or inside*.” |
| **20** | **Strategies to enhance adherence to the study protocols** | Research based strategies that are employed to enhance the delivery of the protocol or the effective use of the wearable by either participants or researchers.  Can include:   - Training of staff, researchers or clinicians - Information booklets for researchers - Information booklets for participants about how the wearable works - Information for participants about why the study is being done - Background information to the study   Strategies may include those that have been implemented in the past or that need to be implemented in the future. | *“And also then to really teach patients when they have to apply this for over several days in their own home that they should take a good look that it is tight enough because of course if it is too loose then you get a lot of noise in your signal, all of those things. I seem to experience that not much of the physiotherapists or also the patients have a good knowledge of that so you really need to educate them.”*  *“I have an information sheet of how to wear and then I go through it with the patient, I go through the importance of it, why we use it, why we need it for seven days, those sorts of things.”* |
| **21** | **The future of wearables** | Researchers opinions on what wearables offer to research in the future (both short and long term).  This includes:  What may develop from wearables in the future  How wearables may change research  How wearables will function in the future  What needs to change for wearables to be useful in the future  What needs to change to reduce current challenges or improve current usage.  How device manufacturers and researchers need to collaborate to improve wearables  **Important:** Overall, this code relates to the ‘big picture’ of wearables in terms of where they are in the context of research overall, rather than specifically looking at the specifications of a particular device  **Do not code** if the researcher is talking about problems or challenges with a specific device that need to be changed. Instead code as ‘barriers and challenges to wearable device use in research” | “*we really need to, it’s a bit more abstract, but we really need to think about how we are going to get people to use these tools in the clinic and how patients are going to use them in the home. What is it that we are going to do that will make it routine practice? What do we have to do to get there?”*  *“it’s a black box. Because they are all very sweet and nice guys but all their validation things if you dig a bit deeper, are very obscure. So I’ve cited this one, they’ve cited me and then we’ve cited each other and this is our validation so it’s like ahhhh cool but I don’t really trust you.”*  *I’m realising this goes more again onto the researcher side of things, is really, I think I’d like to see more, almost more guidance or help, almost from the accelerometer developers in terms of data output and how the researcher can use it right. Like sort of like the whole thing around do you only go off of step count or do you go off the intensity of the physical activity that it tracked, or how do you use it then down the road? Eh, I think that part there is still a lot of room for development.”* |
| **22** | **Usability testing** | Researchers recall of any usability testing they have conducted, including the methods of testing they employed.  Usability in this case refers to “the extent to which a product can be used by specified users to achieve specified goals with effectiveness, efficiency and satisfaction in a specified context of use.”   - Usability testing includes: - Any formal methods that seek to understand this (i.e. questionnaires, interviews, focus groups etc.). - Reasons why usability testing was not completed.   Specific results from usability tests however should be coded as ‘participants opinions regarding acceptability’  **NOTE:** pilot testing within the research group should not be coded as usability, instead code this as ‘personal experience of researchers wearing sensors’ | “*for the most part the teams that we worked with were multi-disciplinary teams so we would have an ethnographer on the team who would look at the overall, yeah, perceived usefulness, perceived ease of use, and we’d use standard usability scales to look at that. And they’d have similar to what we’re doing here, they’d have semi-structured interviews*”  “*we take a lot of time when it comes to deciding what system to use and what we usually do is a dry run where we are wearing the sensors and then if it’s not too bothersome we say ok that’s fine*” |
| **23** | **Other** | Any other code that is seen within the transcripts but does not fit within the parameters and criteria of the above 21 codes, should be coded as ‘other’ |  |
